# Proinflammatory Role of Vascular Endothelial Growth Factor in the Pathogenesis of Rheumatoid Arthritis: Prospects for Therapeutic Intervention

**DOI:** 10.1155/2008/129873

**Published:** 2009-02-10

**Authors:** Seung-Ah Yoo, Seung-Ki Kwok, Wan-Uk Kim

**Affiliations:** Division of Rheumatology, Department of Internal Medicine, School of Medicine, Catholic University of Korea, Seoul 137040, South Korea

## Abstract

Recent experimental and clinical studies have placed new emphasis on the role of angiogenesis in chronic inflammatory disease. Vascular endothelial growth factor (VEGF) and its receptors are the best characterized system in the regulation of rheumatoid arthritis (RA) by angiogenesis. Furthermore, in addition to its angiogenic role, VEGF can act as a direct proinflammatory mediator during the pathogenesis of RA, and protect rheumatoid synoviocytes from apoptosis, which contributes to synovial hyperplasia. Therefore, the developments of synovial inflammation, hyperplasia, and angiogenesis in the joints of RA patients seem to be regulated by a common cue, namely, VEGF. Agents that target VEGF, such as anti-VEGF antibody and aptamer, have yielded promising clinical data in patients with cancer or macular degeneration, and in RA patients, pharmacologic modulations targeting VEGF or its receptor may offer new therapeutic approaches. In this review, the authors integrate current knowledge of VEGF signaling and information on VEGF antagonists gleaned experimentally and place emphasis on the use of synthetic anti-VEGF hexapeptide to prevent VEGF interacting with its receptor.

## 1. INTRODUCTION

The pathology of rheumatoid arthritis (RA) is characterized by the proliferation of synovial cells and angiogenesis, pannus formation. Multiple cell types, including lymphocytes, dendritic cells, macrophages, and synovial fibroblasts, contribute to the chronic inflammatory responses of RA, and comprise a major portion of the invasive pannus [1]. In addition, angiogenesis, the process of new blood vessel formation, is highly active in RA, particularly during the earliest stages of the disease [2, 3]. Newly formed vessels can maintain the chronic inflammatory state by transporting inflammatory cells to sites of synovitis, and supply nutrients and oxygen to the pannus [2, 3]. Angiogenesis is strictly regulated by many inducers and inhibitors, and a number of proangiogenic factors have been suggested to be involved in neovascularization in RA joints. These include acidic and basic fibroblast growth factors, transforming growth factor (TGF)-*β*, angiopoietin, and placenta growth factor (PIGF) in addition to vascular endothelial growth factor (VEGF) [2–4]. 

The final goal of RA treatment is complete disease remission, and not symptomatic relief. At one end of the spectrum of RA treatment outcomes lie a large group of patients who do not respond to single disease-modifying antirheumatic drugs (DMARDs) [5]. Recent clinical trials have suggested that several biologic agents, such as TNF-*α* blockers, rituximab, abatacept, and anakinra, are effective at retarding joint destruction and at alleviating RA activity [5, 6]. However, these biologic agents may have serious side effects, such as predispositions to tuberculosis, lymphoma, progressive multifocal leukoencephalopathy, and high cost, which limit their use [7]. It is also a concern that abrupt stoppages or reductions in these treatments may result in a relapse of disease activity. Moreover, the pathology of RA suggests that it is unlikely that a single biologic agent that targets a specific subset of immune cells is capable of effecting cure.

In this review, we integrate current knowledge concerning how angiogenesis, specifically VEGF, contributes to disease exacerbations in RA. In addition, we present a new therapy for RA based on a synthetic anti-VEGF hexapeptide that specifically targets the interaction between VEGF and its receptor. Prospects for the development of pharmacologic regulators of placental growth factor, which is another angiogenic factor implicated in the pathogenesis of RA, also are discussed.

## 2. PROINFLAMMATORY AND ANTIAPOPTOTIC ROLES OF VEGF IN THE PATHOGENESIS OF RA

VEGF is a dimeric glycoprotein that induces the proliferation and migration of endothelial cells to form new blood vessels, and which increases vascular permeability. VEGF plays important roles during wound healing, embryonic development, the growths of certain solid tumors, and during ascites formation [8]. Several recent reports have demonstrated that VEGF is also implicated in the pathogenesis of RA. Smoking has been recognized as a significant environmental risk factor in RA [9]. Numerous links have been found between cigarette smoking and VEGF [10–12]. VEGF in synovial fluids is significantly more increased in RA than in osteoarthritis [2, 13, 14], and serum levels of VEGF correlate well with RA disease activity, particularly with swollen joint counts [13]. VEGF protein and mRNA are expressed by synovial macrophages and synovial fibroblasts in the synovial tissues of RA patients, and cultured synovial cells are able to secrete VEGF under hypoxic conditions or when stimulated with IL-1, IL-6, IL-17, IL-18, -prostaglandin, or TGF-*β*, or by CD40 ligation [2–4, 15–17]. Furthermore, VEGF knockout mice showed reduced pathology and synovial angiogenesis in antigen-induced models of arthritis [18]. These findings strongly suggest that the inhibition of the angiogenic action of VEGF is likely to suppress rheumatoid inflammation.

Angiogenesis and inflammation are interdependent processes, and inflammatory mediators have significant effects on angiogenesis [2, 3]. Furthermore, recent studies have suggested that the reverse is also true [14, 19]. For example, chronic transgenic delivery of placental growth factor (PlGF) to murine epidermis resulted in a significant increase in inflammatory response [19]. In addition, in a previous study, we demonstrated that 165-amino acid form of VEGF, VEGF_165_, has a direct proinflammatory role in the pathogenesis of RA [14]. In this previous study, recombinant VEGF_165_ was found to increase the productions of TNF-*α* and IL-6 by human peripheral blood mononuclear cells (PBMC). Moreover, the synovial fluid mononuclear cells of RA patients showed a greater response to VEGF_165_ stimulation than the PBMC of healthy controls (the major cell types that responded to VEGF were monocytes). These findings suggest that VEGF_165_ may act as a proinflammatory mediator and as an angiogenic stimulator in RA joints, and thus, they indicate that VEGF is an important link between angiogenesis and the inflammatory process.

**Figure 1:**
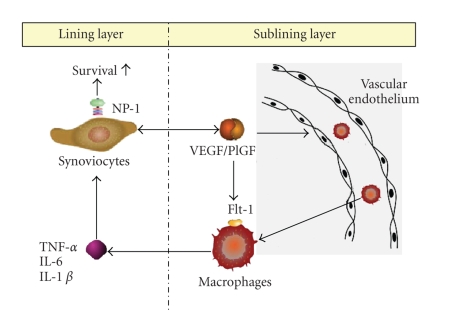
*Scheme for the perpetuation of rheumatoid inflammation by VEGF.* VEGF, which is predominantly produced by rheumatoid synoviocytes, promotes angiogenesis and stimulates vascular endothelial cell permeability. Newly employed macrophages produce TNF-*α* and IL-6 when stimulated by VEGF/Flt-1 binding or by cell contact with activated endothelial cells. TNF-*α* and IL-6, in turn, further enhance the capacity of macrophages and synoviocytes to secrete VEGF, and thus create a self-perpetuating cycle of inflammation. Meanwhile, VEGF binding to NP-1 prevents rheumatoid synoviocytes undergoing apoptosis, which leads to synovial hyperplasia. Hyperplastic synoviocytes, in turn, secrete more VEGF_165_ and by so doing generate a positive feedback-loop that promotes survival. Thus, the development of synovial inflammation, hyperplasia, and angiogenesis in the joints of RA patients may be regulated by a common cue, VEGF.

A number of inflammatory cell types participate in maintaining a mutually activating network in RA joints, which leads to the establishment of a self-perpetuating cycle of autoimmunity [1]. It has been documented that VEGF_165_ activates endothelial cells to produce chemokines, such as MCP-1 and IL-8 [20, 21], which may recruit monocytes around endothelial cells in synovial membranes, where newly employed macrophages, in addition to resident synoviocytes, can produce TNF-*α* and IL-6 when stimulated by VEGF_165_ (as was evidenced by our work) or via cell contact with activated endothelial cells. TNF-*α* and IL-6, in turn, further enhance the capacities of macrophages and synoviocytes to secrete VEGF_165_, and stimulate endothelial cells to induce cell-contact-mediated macrophage activation, which generates a positive feedback-loop (Figure 1). Thus, VEGF_165_ may serve as a functional bridge between endothelial cells and macrophages/synoviocytes.

In RA synovium, synovial fibroblasts proliferate abnormally and invade local environments, and in some ways exhibit the characteristics of tumor cells [22]. Recently, we demonstrated that VEGF is crucially required for the survival of rheumatoid synoviocytes [23]. In this previous study, the ligation of recombinant VEGF_165_ to its receptor prevented synoviocyte apoptosis induced by serum starvation or sodium nitroprusside (SNP). VEGF_165_ rapidly triggered pAkt and pERK activity, and then induced Bcl-2 expression in rheumatoid synoviocytes. Furthermore, VEGF_165_ completely blocked SNP-induced Bcl-2 downregulation and SNP-induced Bax translocation from the cytosol to mitochondria. Collectively, these results suggest that VEGF functions as an important synoviocyte survival factor in RA. As mentioned above, VEGF_165_ is present at higher levels in sera, synovial fluid, and in the inflamed synovial tissues of RA patients than in those of osteoarthritis patients [13, 14]. Therefore, RA synoviocytes are more likely to be stimulated by VEGF_165_ than osteoarthritis synoviocytes, which causes synoviocyte hyperplasia. Moreover, hyperplastic synoviocytes in RA joints secrete more VEGF_165_, and thus, generate positive feedback that promotes their survival (Figure 1).

## 3. EXPRESSION AND FUNCTION OF VEGF RECEPTORS IN RA

VEGF_165_ exerts its biological effects by binding with its receptor subtypes, that is, fms-like tyrosine kinase (Flt-1), kinase insert domain-containing receptor (KDR) and neuropilin-1 (NP-1) [3]. Flt-1 and KDR exhibit tyrosine kinase activity, and both are expressed in the majority of vascular endothelial cells [8, 24, 25]. KDR is a primary mediator of endothelial cell proliferation in response to VEGF_165_, whereas unlike KDR, Flt-1 is present in inflammatory cells, such as, macrophages and monocytes [8, 24, 25]. Therefore, in addition to its proangiogenic action, Flt-1 is critically involved in monocyte activation, and in addition, it also promotes the mobilization of myeloid progenitors from bone marrow to the blood [8, 24, 25]. On the other hand, NP-1 has been demonstrated to function as a nontyrosine kinase receptor for VEGF_165_, and specifically, for the heparin-binding domain of VEGF_165_ [26, 27]. NP-1 was initially characterized as a receptor for semaphorin 3A, which mediates the guidance of neuronal cells [28]. In endothelial cells, NP-1 is also a coreceptor of VEGF, and has been shown to regulate KDR-dependent angiogenesis [26, 27]. Furthermore, NP-1 mediates the antiapoptotic activity of VEGF_165_ in breast cancer cells [29].

All three of VEGF_165_ receptor subtypes are expressed in RA synovium [30]. We previously demonstrated that NP-1, rather than Flt-1 or KDR, is the major VEGF_165_ receptor in RA synoviocytes [23]. NP-1 was found to be highly expressed in the lining layer, and on infiltrating leukocytes and endothelial cells of the rheumatoid synovium. Furthermore, the downregulation of NP-1 transcripts by short interfering RNA caused spontaneous synoviocyte apoptosis, which was associated with both a decrease in Bcl-2 expression and an increase in Bax translocation to mitochondria. Therefore, NP-1 appears to play a critical role in maintaining RA synoviocyte survival. In addition, more recently, we found that Flt-1 is highly expressed in the sublining of leukocytes in RA synovium, and in monocytes from the PBMC of active RA patients [31]. Furthermore, Flt-1 expression levels were found to be well correlated with erythrocyte sedimentation rates, a marker of disease activity, indicating that they reflect inflammatory activity of RA [31]. These findings suggest that chronic inflammatory milieux, such as those generated by high concentrations of proinflammatory cytokines, may upregulate Flt-1 expression on RA monocytes.

There are several potential mechanisms whereby VEGF receptors could contribute to RA inflammation. First, VEGF_165_ binding to KDR may lead to an increase in angiogenesis, and thereby, the recruitment of peripheral leukocytes to inflamed synovium, which diminishes the growing burden of synoviocytes by supplying the oxygen and nutrients required for tissue metabolism. Second, via interaction with Flt-1, VEGF_165_ may directly stimulate the productions of cytochemokines, such as TNF-*α*, IL-6, MCP-1, and IL-8, which are essential for the perpetuation of chronic inflammation in joints. Third, NP-1 could hamper synoviocyte apoptosis upon ligation of VEGF_165_, and thus, function as a survival factor, in an autocrine or paracrine fashion. In this manner, VEGF_165_ would simultaneously regulate the developments of synovial inflammation, hyperplasia, and angiogenesis in RA joints (Figure 1).

## 4. BLOCKADE OF VEGF AND ITS RECEPTOR IN RA

The success of anti-VEGF antibody (Ab) treatment in cancer patients raises the possibility of applying antiangiogenic therapies in other diseases, such as retinopathy, RA, and other inflammatory disorders. Given the pleiotropic roles played by VEGF and its receptor in RA inflammation [2, 3, 8, 24], it can be postulated that anti-VEGF treatment retards chronic synovitis in several ways, as follows: (a) it may decrease nutrient supply to the tumor-like synovium; (b) inhibit leukocyte adhesion and migration by decreasing endothelial cell surface area; (c) decrease chemokine and cytokine productions by activated endothelial cells; (d) reduce the VEGF-induced productions of TNF-*α* and IL-6 by monocytes/macrophages; (e) abrogate VEGF-induced increases in synoviocyte survival. These different mechanisms may occur independently, but it remains to be determined which mechanism plays a dominant role in the quenching of RA inflammation.

Bevacizumab, a humanized form of anti-VEGF Ab, was the first antiangiogenic agent approved by the FDA in the US for the treatment of metastatic colon cancer [32], and it was also found to be beneficial for the treatment of lung and renal cell cancer [33, 34]. Although bevacizumab is generally well tolerated, it has some serious toxic effects, for example, hypertension, bleeding, and arterial thromboembolism, which occur infrequently [32–34]. Currently, various different developmental approaches to inhibit VEGF and its receptors are in progress. The FDA approved pegaptanib, an anti-VEGF RNA aptamer, for the treatment of neovascular age-related macular degeneration (AMD) [35]. Notably, pegaptanib was found to reduce vision loss in AMD patients by about 50% during the first treatment year and to stabilize vision during the second year. Other agents which target VEGF receptors, such as, chimerized anti-KDR antibody, VEGF-Trap, and a synthetic ribozyme of Flt-1, are also undergoing phase I or II trials for the treatment of solid tumors and cancer [36].

The effects of VEGF and of its receptor antagonists have also been tested in experimental models of RA. Neutralizing Ab to VEGF was found to prevent collagen-induced arthritis and to ameliorate established disease in mice [37], and treatment with a soluble form of Flt-1 receptor significantly attenuated the severity of murine collagen-induced arthritis [38]. Interestingly, the failure of anti-KDR Ab, but not of anti-Flt-1 Ab, to block arthritis and atherosclerosis [24, 25, 39], indicates that anti-inflammation, rather than antiangiogenesis, may be primarily responsible for the observed effects of anti-VEGF Ab. Considering that Flt-1 tyrosine kinase signaling promotes RA via monocyte/macrophage activation [31, 40], the selective inhibition of Flt-1 may be effective at blocking VEGF-induced inflammation and angiogenesis with minimal toxicity. However, no clinical trial on anti-VEGF inhibitors has been undertaken in RA. Indeed, some antirheumatic drugs with well-known clinical efficacy in RA, such as cyclosporin and anti-TNF-*α* Ab, have been reported to inhibit VEGF production in RA patients [41, 42].

Through the screening of positional scanning synthetic peptide libraries, we identified a soluble arginine-rich hexapeptide sequence, RRKRRR (Arg-Arg-Lys-Arg-Arg-Arg), which binds to VEGF_165_, and thereby prevents it from interacting with its receptor [14]. To increase the in vivo stability of this peptide, we changed its peptide structure from the L- to the D-form, and accordingly, were able to increase its half life to more than 24 hours [14], which makes the peptide more suitable for therapeutic applications. In mice, the hexapeptide RRKRRR significantly inhibited VEGF-induced angiogenesis, and also retarded the growth and metastasis of colon carcinoma cells. In addition, it strongly inhibited ongoing paw inflammation in arthritic mice without apparent side effects [14]. When compared with several known VEGF antagonists, such as anti-VEGF antibody and aptamer, RRKRRR is advantageous from the clinical standpoint because it is a short peptide that is easily synthesized and because it has low immunogenicity. In a similar manner, Bae et al., also found that the novel anti-Flt-1 hexapeptide, GNQWFI (Gly-Asn-Gln-Trp-Phe-IIe), inhibited angiogenesis and tumor growth without side effects [43]. This peptide selectively binds to Flt-1, and thereby, blocks the interaction between Flt-1 and VEGF or PlGF. Investigations on the effect of anti-Flt-1 peptide GNQWFI on an experimental model of arthritis are under way.

## 5. PLACENTAL GROWTH FACTOR: A NEW POTENTIAL TARGET FOR ANTIANGIOGENIC TREATMENT

PlGF is a member of the VEGF family, which was first identified in placenta, but is also known to be present in heart, lung, and joints [44]. As a specific ligand for Flt-1, PlGF has potent angiogenic properties, and it also induces the growth and migration of endothelial cells [24, 25]. In addition, PlGF stimulates tissue factor production and chemotaxis in monocytes [45], and also increases TNF-*α*, IL-1, IL-6, IL-8, and MCP-1 productions by normal and/or rheumatoid monocytes [31, 46], which suggests that it directly modulates the inflammatory process. PlGF concentrations were reported to be increased in RA synovial fluids, and to induce VEGF production by mononuclear cells [47]. Moreover, genetic ablation of PlGF prevented the development of antibody-induced arthritis in mice [31] suggesting the critical role of PlGF in RA inflammation.

PlGF exhibits functions that are distinct from those of VEGF, in that it regulates the angiogenic switch during the diseased state [48]. It was recently reported that neutralizing Ab to PlGF inhibits the growth and metastasis of various tumors, including those resistant to VEGF inhibitors, and that it enhances the efficacies of chemotherapy and that of anti-KDR Ab [48]. Unlike anti-KDR Ab, anti-PlGF Ab prevented the infiltration of angiogenic macrophages and severe tumor hypoxia, and thus, did not switch on the angiogenic rescue program responsible for resistance to anti-KDR Ab [48]. Furthermore, it did not cause or enhance anti-KDR Ab-related side effects, such as, the inhibition of placental vascular development. Similar suppressive effects of PlGF antagonist on tumor growth were observed in another study, in which an antagonistic PlGF peptide (SHRYRLAIQLHASDSSSSCV) inhibited the growth and metastasis of human breast cancer xenografts [49]. Taken together, PlGF antagonists may prevent angiogenesis and tumor growth without affecting normal physiology, and thus, are ideal candidates for RA treatment. We are currently investigating whether synthetic anti-PlGF peptides inhibit the severity of arthritis and angiogenesis.

## 6. CONCLUSION

We and others have demonstrated that proangiogenic factors, such as VEGF and PlGF, exert direct proinflammatory [14, 19–21, 31, 40, 45, 46] and antiapoptotic effects [23, 29]. In this regards, the developments of synovial inflammation, hyperplasia, and angiogenesis in the joints of RA patients may all be regulated by VEGF. Given the importance of VEGF in the pathology of RA, antiangiogenic therapies, particularly those involving an anti-Flt-1 blocking agents, could when administered as a monotherapy or in combination with other biologic agents selectively ameliorate RA symptoms and reverse its fundamental pathology. The antiangiogenic peptides, RRKRRR and GNQWFI, introduced here represent a promising development in the antiangiogenic field. Hopefully, our efforts will result in clinical applications.
